# DZ-1-artesunate conjugate induces mitochondria-mediated, reactive oxygen species-dependent apoptosis in colorectal cancer tumoroids

**DOI:** 10.3389/fphar.2026.1743141

**Published:** 2026-03-20

**Authors:** Badrinath Narayanasamy, Yi Zhang, Alexandra Gangi, Robert Figlin, Karine Sargsyan, Heuiran Lee, Cheryn Song, Yong J. Lee

**Affiliations:** 1 Cedars-Sinai Cancer Institute and Department of Biomedical Sciences, Cedars-Sinai Medical Center, Los Angeles, CA, United States; 2 Cedars-Sinai Cancer Institute and Department of Surgery, Cedars-Sinai Medical Center, Los Angeles, CA, United States; 3 Cedars-Sinai Cancer Institute and Division of Hematology, Cedars-Sinai Medical Center, Los Angeles, CA, United States; 4 Bio-Medical Institute of Technology, College of Medicine, University of Ulsan, Seoul, Republic of Korea; 5 Department of Microbiology, Asan Medical Center, College of Medicine, University of Ulsan, Seoul, Republic of Korea; 6 Department of Urology, Asan Medical Center, College of Medicine, University of Ulsan, Seoul, Republic of Korea

**Keywords:** apoptosis, artesunate, DZ-1, mitochondria, oxidative stress, patient-derived tumoroids, reactive oxygen species

## Abstract

We previously reported that the heptamethine cyanine dye–conjugated artesunate (DZ-1-ART) induces apoptosis in monolayer (2D) cell culture models. However, in 2D cultures, cells grow on flat, rigid plastic surfaces that fail to recapitulate the three-dimensional architecture of tumor tissues. This artificial environment alters cell polarity, morphology, and mechanical signaling, leading to non-physiological behavior. To overcome these limitations, we developed patient-derived tumoroids to assess the tumoricidal efficacy of the DZ-1-ART conjugate. In this study, tumoroids were established from fresh tissue samples of a malignant neoplasm of the sigmoid colon. The anticancer activity of DZ-1-ART was evaluated in these tumoroids. Propidium iodide staining confirmed DZ-1-ART–induced cytotoxicity, while TUNEL and immunoblotting assays demonstrated that this cytotoxicity was mediated by apoptosis. Furthermore, MitoTracker staining and near-infrared fluorescence indicated mitochondrial localization of DZ-1-ART. The JC-1 assay showed disruption of mitochondrial membrane potential following DZ-1-ART treatment. Additionally, deferoxamine and MitoTEMPO pretreatment revealed that DZ-1-ART induced mitochondria-mediated reactive oxygen species (ROS) generation in tumoroids. Collectively, these findings suggest that DZ-1-ART acts as a potent mitochondria-targeting anticancer agent with potential for precision therapy.

## 1 Introduction

In the United States, colorectal cancer (CRC) is being the third most diagnosed cancer and a significant health issue in globally (Siegel et al., 2023; Matsuda et al., 2025). The age, genetics and inflammatory bowel disease are some of the key risk factors of CRC. The lifestyle activities, such as obesity, low physical activity, active and passive smoking, high salt and red meat consumption have also been linked with an increased risk of colorectal cancer (Johnson et al., 2013; Lewandowska et al., 2022).

The current therapeutic approaches for CRC are surgical removal of resectable CRC, radiotherapy chemotherapy, immunotherapy, and their combinational treatment for non-resectable CRC (Kumar et al., 2023). However, limitations like drug resistance, systemic toxicity and nonspecific targeting diminish therapeutic efficacy (Xie et al., 2020; Ashique et al., 2024). Historically, *in vitro* systems such as immortalized cancer cell lines have been widely used to study cancer therapeutics due to their robustness and tractability. However, these models have critical limitations: their phenotypic and molecular characteristics are often altered by adaptation to artificial culture conditions, leading to cellular behaviors that diverge substantially from those of primary tumors (Pandita et al., 2004; De Witt Hamer et al., 2008). Moreover, targeted therapy for CRC is being developed (Ruff et al., 2023). Patient derived xenograft (PDX) and tumoroids are developed for precision therapy (Kim et al., 2025). Over the PDX, tumoroids can represent genetic diversity, cellular and pathophysiological characteristics of original tumors (Luo et al., 2024).

Researchers have developed DZ-1, a near-infrared (NIR) heptamethine cyanine (HMCD) dye, primarily as a tumor-targeting imaging agent. DZ-1 is selectively taken up by cancer cells rather than normal tissues, with tumor specificity largely attributed to the overexpression of organic anion transporting polypeptides (OATPs) in malignant cells (Wu et al., 2015; Schulte and Ho, 2019; Mrdenovic et al., 2023). Conjugation of therapeutic agents to imaging dyes can alter bioavailability, pharmacokinetics, and clearance, although the magnitude and direction of these effects depend on multiple factors, including the chemical structure of the conjugated drug, the linker composition, and the route of administration (Torchilin, 2006; Soo Choi et al., 2007; Smith et al., 2008; Liu et al., 2009). Previous studies demonstrated that DZ-1–gemcitabine conjugates retained tumor-specific targeting, exhibited improved therapeutic bioavailability, reduced systemic clearance, and minimized off-target distribution (Zhang et al., 2017). Building on these findings, we developed a tumor-targeting DZ-1–artesunate conjugate (DZ-1-ART) to preferentially deliver artesunate to tumor tissues.

In this study, we successfully cultured a patient-derived CRC tumoroids and treated with DZ-1-ART. We evaluated the tumor-targeting efficacy of DZ-1-ART in these CRC tumoroids. Notably, our results demonstrated mitochondrial localization of DZ-1-ART and subsequent ROS generation. Furthermore, ROS production was associated with apoptosis mediated via the caspase signaling pathway in DZ-1-ART–treated tumoroids.

## 2 Materials and methods

### 2.1 Tumor tissue isolation

Fresh tissue samples from a malignant neoplasm of the sigmoid colon were obtained from a low anterior resection performed by Dr. Alexandra Gangi at Cedars-Sinai Medical Center (IRB approval STUDY00002365). Tumor specimens were stored in Servator B Storage Solution in a 50 mL tube at 4 °C for up to 24 h prior to processing the following day.

To isolate tumor cells, tissue samples were first transferred into a Petri dish containing ice-cold phosphate-buffered saline (PBS) without Ca^2+^ and Mg^2+^ using sterile forceps and washed with cold DPBS to remove blood and debris. Tumor tissues were then minced into small pieces and transferred onto a cell strainer placed over a 50 mL tube. Using a 5 mL syringe plunger, the tissue fragments were further dissociated and filtered through the strainer, followed by centrifugation at 1,000 × g for 5 min. The resulting pellet was resuspended in 5 mL of ACK Blood Lysis Buffer and incubated at room temperature for 15 min. After incubation, samples were centrifuged at 1,000 × g for 5 min. This lysis step was repeated until the tissue appeared white, indicating effective removal of red blood cells.

Fresh tissue samples were washed three times with a freshly prepared 2 mM ethylenediaminetetraacetic acid (EDTA) chelation buffer and incubated on ice for 1 h on a horizontal orbital shaker. Following incubation, tissues were centrifuged and washed three times with ice-cold chelation buffer without EDTA. The tissue pellets were then resuspended in 15 mL of digestion buffer and transferred to a T25 flask for a 2-h incubation at 37 °C. The resulting suspension was filtered through a small strainer into a 50 mL tube and briefly centrifuged. The final pellet was resuspended in ice-cold chelation buffer and used for subsequent tumoroid culture.

### 2.2 Tumoroid formation establishment

The complete cell culture medium (DMEM/F-12 + GlutaMAX supplemented with R-spondin 1 [500 ng/mL], Noggin [100 ng/mL], EGF [50 ng/mL], A83-01 [0.2 µM], Y-27632 dihydrochloride [10 µM], B27 [50X], and N-acetylcysteine [1.25 mM]) was mixed with Matrigel at a 1:2 ratio. Isolated tumor tissues were resuspended in the Matrigel–medium mixture and filtered through a Flowmi® Cell Strainer into a clean 15 mL tube. For seeding, 100 μL of the suspension was pipetted into the center of each well of a pre-incubated 12-well plate. The plate was incubated at 37 °C in an inverted position for 15 min, followed by an additional 5-min incubation in the standard orientation to allow gel polymerization. For tumoroid formation, 1 mL of complete cell culture medium was carefully added to each well, and the cultures were maintained at 37 °C in a 5% CO_2_ incubator.

### 2.3 Tumoroids seeding for the treatment

After successful formation, tumoroids were harvested from the 12-well plates and centrifuged at 1,000 × g for 5 min, and the supernatant was discarded. The tumoroid pellets were incubated with 1 mL of trypsin for 5 min at 37 °C in a 5% CO_2_ incubator. Following digestion, tumoroids were resuspended in 2 mL of complete medium, centrifuged again at 1,000 × g for 5 min, and the supernatant was removed. The pellets were then resuspended in a Matrigel–complete medium mixture and filtered through a Flowmi® Cell Strainer. For reseeding, 100 μL of the filtered tumoroid–Matrigel suspension was pipetted into the center of each well of a 12-well plate, which was incubated inversely at 37 °C for 15 min. Finally, 1 mL of complete medium was carefully added to each well for continued culture.

### 2.4 Chemical and reagents

Deferoxamine (DFO) mesylate salt (Cat# D9533), N-acetylcysteine (NAC) (Cat# A7250), 2′,7′-dichlorofluorescin diacetate (DCFDA) (Cat# D6883), and MitoTEMPO (Cat# SML0737) were purchased from Millipore Sigma. DZ-1 and DZ-1–artesunate (DZ-1-ART) conjugates were synthesized by Dr. Yi Zhang at Cedars-Sinai Medical Center (Los Angeles, CA).

### 2.5 Trypan blue exclusion assay for half-maximal inhibitory concentration (IC50) determination

Tumoroids were seeded in 12-well plates using Matrigel, followed by the addition of complete culture medium. Two-fold serial dilutions of DZ-1-ART were applied, and cells were treated for 24 h. For the trypan blue exclusion assay, DZ-1-ART–treated tumoroids were harvested and dissociated with trypsin for cell viability assessment. Cells were stained with 0.4% trypan blue, and viable (unstained) cells were counted. The IC_50_ value was determined by nonlinear regression analysis using GraphPad Prism.

### 2.6 Immunoblotting and antibodies

Immunoblotting was performed as previously described (Nam et al., 2002). Primary antibodies, such as anti-PARP-1 (Cat #9532), anti-caspase-8 (Cat #9746), anti-cleaved caspase-9 (Cat#7237), anti-ATF-4 (Cat#11815S), anti-BiP (Cat#3177S) and anti-HO-1 (Cat#5061S) were purchased from Cell Signaling Technology. Anti-β-actin (Cat #A1978) was purchased from Sigma-Aldrich. For secondary antibodies, anti-rabbit IgG-HRP (Cat#7074P2) and goat anti-mouse IgG-HRP were obtained from Cell Signaling Technology and Santa Cruz Biotechnology, respectively.

### 2.7 Propidium iodide staining

Tumoroids were seeded in 12-well plates and treated for the indicated durations. Following treatment, cells were washed with cold PBS and dissociated using 0.25% trypsin-EDTA. The trypsinized cells were resuspended in complete medium and centrifuged at 1,000 × g for 5 min at 4 °C. The supernatant was discarded, and the cell pellet was washed with ice-cold PBS, followed by a second centrifugation under the same conditions. The resulting cells were resuspended in binding buffer and stained with propidium iodide (PI) for 10 min at room temperature. After incubation, cells were washed with PBS and counterstained with Hoechst for 10 min. Fluorescence images were acquired using an ECHO fluorescence microscope.

### 2.8 TUNEL assay

Apoptosis was evaluated using the terminal deoxynucleotidyl transferase dUTP nick end labeling (TUNEL) assay. Tumoroids were treated with DZ-1-ART for the indicated time intervals and then washed with 1× PBS. The *In Situ* Cell Death Detection Kit, Fluorescein (Roche, Cat# 11684795910) was used according to the manufacturer’s instructions. Briefly, the TUNEL reaction mixture was added to the tumoroids and incubated at 37 °C for 1 h, followed by PBS washing and Hoechst counterstaining for 10 min. Fluorescence images were acquired using an ECHO fluorescence microscope.

### 2.9 Mitochondria labeling

Mitochondria were labeled using the MitoTracker™ Green FM kit (Cat# M7514) according to the manufacturer’s instructions. After 4, 8, 16, and 24 h of DZ-1-ART treatment, tumoroids were incubated with MitoTracker Green FM dye. Hoechst staining was performed to visualize nuclei. Fluorescence images were captured using an ECHO fluorescence microscope to detect green (mitochondrial) and blue (nuclear) signals.

### 2.10 JC-1 assay

Mitochondrial membrane potential (ΔΨm) was assessed using the JC-1 dye (Thermo Fisher Scientific, Cat# T3168). After 4, 8, 16, and 24 h of DZ-1-ART treatment, tumoroids were stained with JC-1 according to the manufacturer’s instructions. Fluorescence images were captured using an ECHO fluorescence microscope to detect red (J-aggregates) and green (monomers) signals indicative of mitochondrial polarization status.

### 2.11 ROS assay

Tumoroids were treated with DZ-1-ART in the presence or absence of N-acetylcysteine (NAC). ROS generation was evaluated using the DCFDA assay. After 4, 8, 16, and 24 h of treatment, tumoroids were incubated with DCFDA according to the manufacturer’s instructions. Following 45 min of incubation, Hoechst staining was performed to visualize nuclei, and fluorescence images were acquired using an ECHO fluorescence microscope.

### 2.12 MitoSOX assay

Tumoroids treated with DZ-1-ART in the presence of DFO or MitoTEMPO for 24 h were stained with MitoSOX™ Red mitochondrial superoxide indicator (Thermo Fisher Scientific, Cat# 36008). After 1 h of incubation, tumoroids were counterstained with Hoechst, and fluorescence images were captured using an ECHO fluorescence microscope.

### 2.13 Statistical analysis

Statistical analysis was performed using one-way analysis of variance (ANOVA) followed by Tukey’s *post hoc* test. All analyses were conducted using GraphPad Prism nine software. Data are presented as mean ± standard deviation (SD). Differences were considered statistically significant at P < 0.05, with significance indicated as: *P < 0.05, **P < 0.01, ***P < 0.001, and ****P < 0.0001.

## 3 Results

### 3.1 DZ-1-ART induces tumoroidal toxicity in patient-derived tumoroids

**FIGURE 1:**
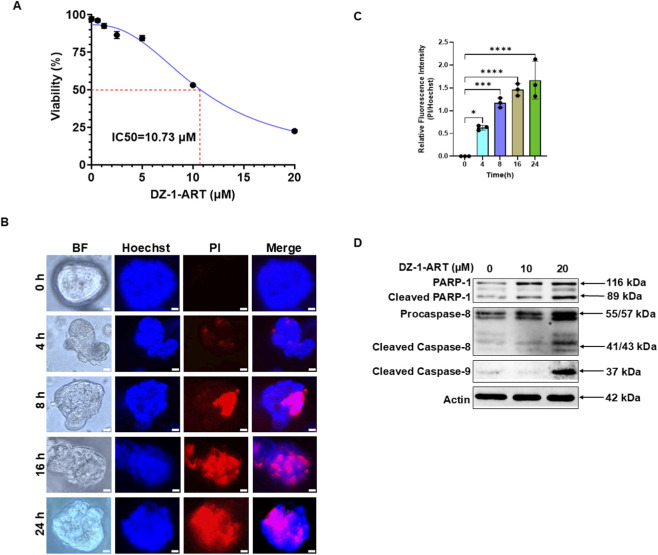
DZ-1-ART induces tumoroidal toxicity in colon cancer tumoroids. **(A)** To determine the IC50 of DZ-1-ART, tumoroids were treated with two-fold serial dilutions of DZ-1-ART for 24 h. Cell viability was assessed using the trypan blue exclusion assay. The IC_50_ value was calculated using nonlinear regression curve analysis. **(B)** Tumoroids were treated with 10 µM DZ-1-ART for 4, 8, 16, and 24 h. Cytotoxicity and nuclear localization were assessed by staining with propidium iodide (PI) and Hoechst, respectively. Images were captured using ECHO fluorescence microscopy. **(C)** Relative fluorescence intensity of the PI/Hoechst ratio in DZ-1-ART–treated tumoroids at different time points. Data were analyzed using one-way ANOVA followed by Tukey’s multiple comparison test (*P < 0.05, **P < 0.01, ***P < 0.001, ****P < 0.0001). Error bars represent mean ± SD of triplicates. **(D)** Tumoroids were treated with 10 µM or 20 µM DZ-1-ART for 24 h. Cells were lysed with 1× lysis buffer, and protein concentrations were quantified using the BCA assay. Proteins were separated by SDS-PAGE and analyzed by Western blotting using the indicated antibodies.

Patient-derived tumoroids were seeded in 12-well plates to evaluate the tumoroidal toxic effect of DZ-1-ART. To determine the IC_50_ concentration of DZ-1-ART in tumoroids, two-fold serial dilutions (20, 10, 5, 2.5, 1.25, and 0.62 µM) were prepared and applied to the cultures. After 24 h of treatment, cell viability was assessed using the trypan blue exclusion assay. Nonlinear regression curve analysis revealed an IC50 value of 10.73 µM for DZ-1-ART (Figure 1A). Tumoroids were treated with 10 µM DZ-1-ART for 4, 8, 16, and 24 h. At each time point, tumoroids were stained with propidium iodide (PI) to assess tumoroidal toxicity. The results demonstrated a clear time-dependent increase in tumoroidal toxicity. PI staining showed that tumoroidal toxicity became detectable at 4 h, increased substantially by 8 h, reached approximately 80% at 16 h, and approached nearly complete (≈100%) death by 24 h (Figures 1B,C).

### 3.2 Apoptosis mediates DZ-1-ART-induced tumoroidal toxicity in patient-derived tumoroids

**FIGURE 2:**
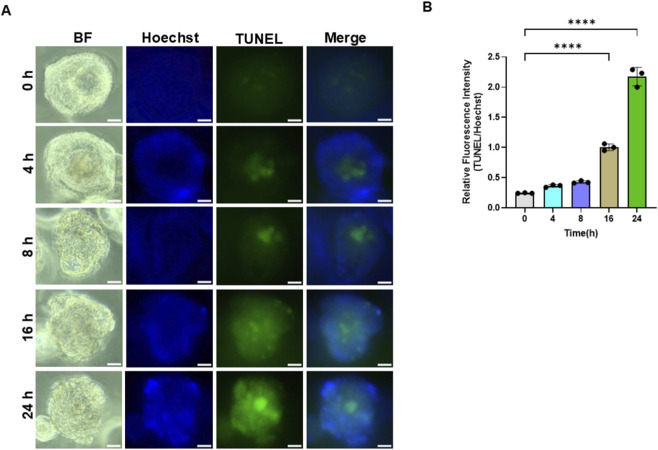
DZ-1-ART induces apoptosis in colon cancer tumoroids. **(A)** Apoptosis in tumoroids was assessed using the TUNEL assay. Tumoroids were treated with 10 µM DZ-1-ART for 4, 8, 16, and 24 h and stained with TUNEL and Hoechst to detect apoptotic cells and nuclei, respectively. TUNEL fluorescence images were captured using ECHO fluorescence microscopy. **(B)** Relative fluorescence intensity of the TUNEL/Hoechst ratio in DZ-1-ART–treated tumoroids at different time points. Data were analyzed by one-way ANOVA followed by Tukey’s multiple comparison test (****P < 0.0001).

To determine the mechanism underlying DZ-1-ART–induced tumoroidal toxicity, patient-derived tumoroids were treated with 10 µM or 20 µM DZ-1-ART for 24 h. Following treatment, tumoroids were collected and lysed for immunoblotting analysis. Cleavage (activation) of poly (ADP-ribose) polymerase-1 (PARP-1), caspase-8, and caspase-9 in DZ-1-ART–treated samples confirmed that apoptosis mediates DZ-1-ART–induced tumoroidal toxicity (Figure 1D). Consistently, TUNEL assays performed at different time intervals further validated the involvement of apoptosis in DZ-1-ART–induced tumoroidal toxicity (Figures 2A,B).

### 3.3 DZ-1-ART targets mitochondria in patient-derived tumoroids

**FIGURE 3:**
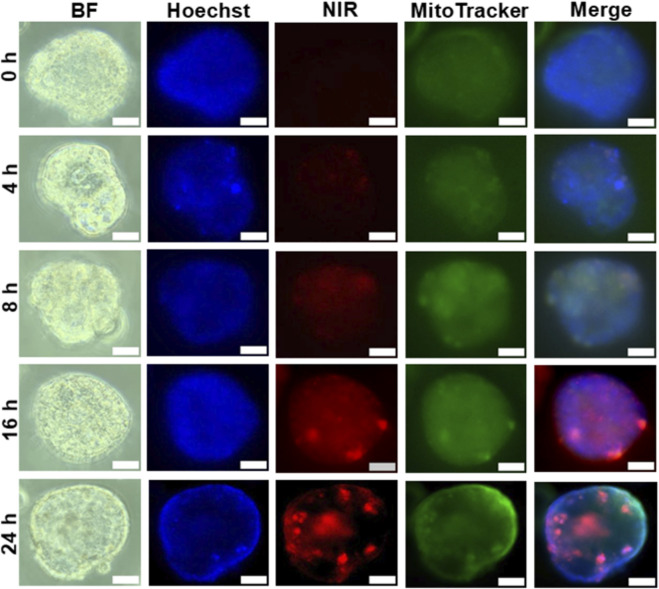
Mitochondrial localization of DZ-1-ART in colon cancer tumoroids. Tumoroids were treated with 10 µM DZ-1-ART for 4, 8, 16, and 24 h, then stained with MitoTracker to label mitochondria and Hoechst to label nuclei. Green fluorescence representing DZ-1-ART localization was captured using ECHO fluorescence microscopy.

To investigate the cellular uptake and subcellular localization of DZ-1-ART, patient-derived tumoroids were treated with 10 µM DZ-1-ART for various time intervals and subsequently stained with MitoTracker Green. NIR fluorescence imaging revealed colocalization of DZ-1-ART signals with mitochondria. After 24 h of treatment, DZ-1-ART predominantly accumulated within mitochondria (Figure 3).

### 3.4 DZ-1-ART disrupts mitochondrial membrane potential in patient-derived tumoroids

**FIGURE 4:**
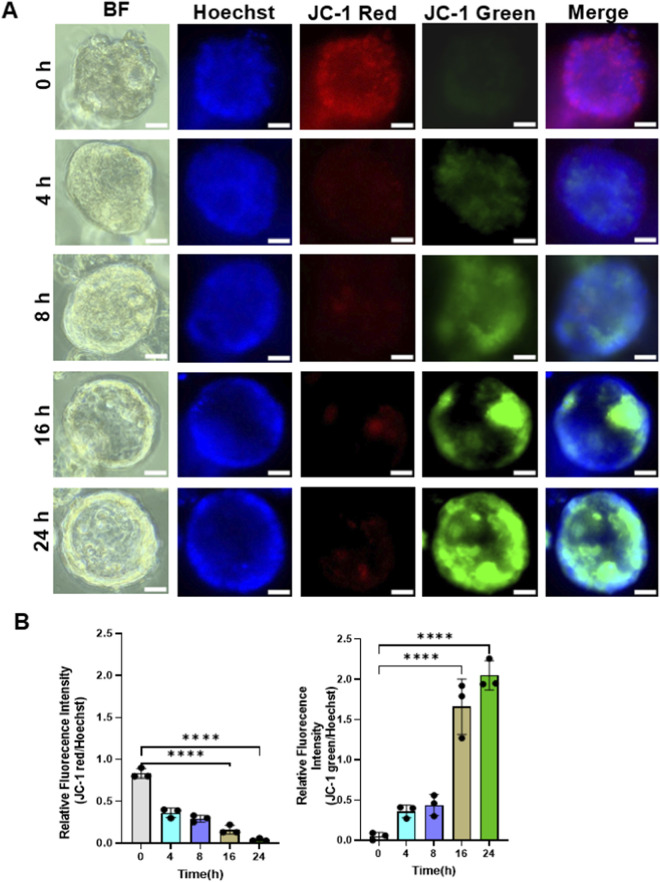
DZ-1-ART disrupts mitochondrial membrane potential in colon cancer tumoroids. **(A)** Tumoroids were treated with 10 µM DZ-1-ART for 4, 8, 16, and 24 h, stained with JC-1 to assess mitochondrial membrane potential, and Hoechst to label nuclei. Fluorescence images were captured using ECHO fluorescence microscopy. **(B)** Relative fluorescence intensity of the JC-1 red/green ratio in DZ-1-ART–treated tumoroids at different time points. Data were analyzed using one-way ANOVA followed by Tukey’s multiple comparison test (****P < 0.0001).

Since DZ-1-ART was localized within mitochondria, we next examined whether it affects mitochondrial membrane potential (ΔΨm). Tumoroids were treated with DZ-1-ART for the indicated time intervals and subsequently stained with JC-1 dye. The results showed that DZ-1-ART induced a time-dependent disruption of ΔΨm, which became detectable at 4 h and markedly increased at 16 and 24 h (Figures 4A,B).

### 3.5 DZ-1-ART induced disruption ΔΨm generates ROS in patient-derived tumoroids

**FIGURE 5:**
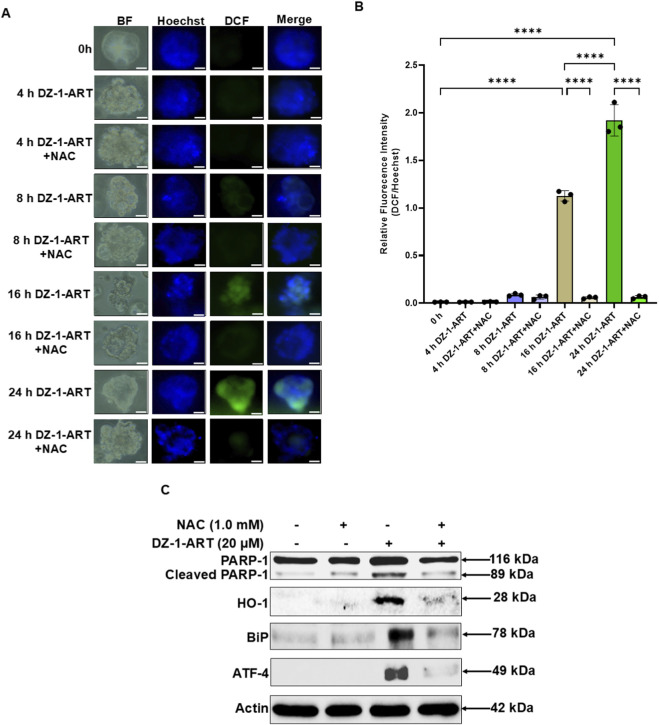
DZ-1-ART induces ROS generation, which is suppressed by NAC. **(A)** Tumoroids were treated with 10 µM DZ-1-ART with or without 1 mM NAC for indicated time intervals. Following treatment, tumoroids were incubated with DCFDA for 45 min and stained with Hoechst to label nuclei. Fluorescence images were captured using ECHO fluorescence microscopy. **(B)** Relative fluorescence intensity of the DCF/Hoechst ratio in DZ-1-ART ± NAC–treated tumoroids. Data were analyzed by one-way ANOVA followed by Tukey’s multiple comparison test (****P < 0.0001). **(C)** Tumoroids were treated with 10 µM DZ-1-ART in the presence or absence of 1 mM NAC for 24 h and then harvested for immunoblot analysis using the indicated antibodies.

Disruption of mitochondrial membrane potential (ΔΨm) by DZ-1-ART can lead to mitochondrial ROS production. To investigate this possibility, tumoroids were treated with DZ-1-ART, with or without the ROS scavenger N-acetyl cysteine (NAC), for different time intervals. ROS levels were assessed using DCF staining. After 16 h of DZ-1-ART treatment, increased ROS production was detected, which was effectively inhibited by NAC. At 24 h, DZ-1-ART induced robust ROS generation, whereas NAC treatment suppressed ROS accumulation (Figures 5A,B).

### 3.6 DZ-1-ART also induces apoptosis endoplasmic reticulum (ER)-associated oxidative stress in patient-derived tumoroids

To investigate the effect of DZ-1-ART on ER-associated oxidative stress, tumoroids were treated with 10 µM DZ-1-ART in the presence or absence of NAC. After 24 h, tumoroids were harvested, and the protein expression levels of oxidative stress markers, including Activating transcription factor 4 (ATF-4), binding immunoglobulin protein (BiP), and heme oxygenase-1 (HO-1) were analyzed. The results showed upregulated expression of these proteins in DZ-1-ART–treated tumoroids, whereas their expression was downregulated upon NAC co-treatment (Figure 5C). Collectively, these findings confirm the involvement of ER-associated oxidative stress in DZ-1-ART–induced apoptosis.

### 3.7 MitoTEMPO confirms DZ-1-ART-induced mitochondrial ROS in patient-derived tumoroids

**FIGURE 6:**
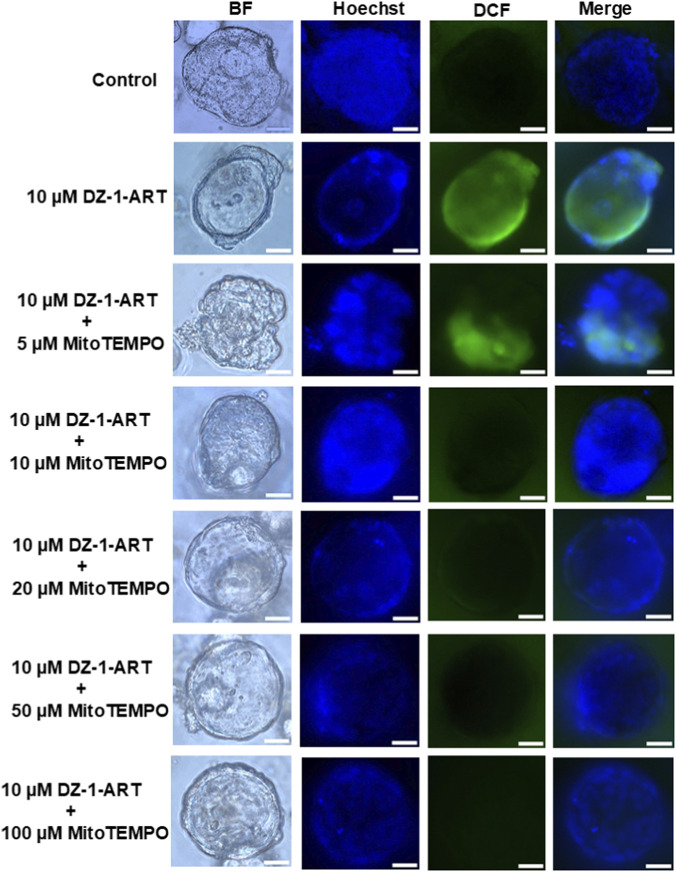
MitoTEMPO suppresses DZ-1-ART–induced ROS generation. Tumoroids were treated with 10 µM DZ-1-ART in the presence of varying concentrations of MitoTEMPO for 24 h. ROS generation was assessed by incubating the treated tumoroids with DCFDA, and green fluorescence was captured using ECHO fluorescence microscopy.

MitoTEMPO is a mitochondria-targeted antioxidant that scavenges mitochondrial superoxide (Shetty et al., 2021). To confirm that DZ-1-ART generates ROS specifically from mitochondria, tumoroids were treated with 10 µM DZ-1-ART in the presence of varying concentrations of MitoTEMPO. DCF staining showed that co-treatment with 10 µM MitoTEMPO markedly reduced DZ-1-ART–induced green fluorescence, indicating effective inhibition of ROS (Figure 6). These results confirm that DZ-1-ART induces ROS production within mitochondria in patient-derived tumoroids.

### 3.8 DZ-1-ART induces Fenton-reaction mediated ROS and apoptosis in patient-derived tumoroids

**FIGURE 7:**
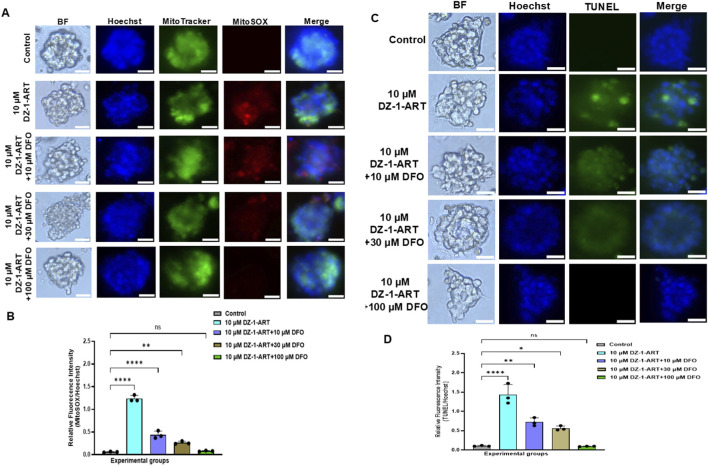
DZ-1-ART induces mitochondria mediated iron dependent ROS and apoptosis. **(A)** Tumoroids were treated with 10 µM of DZ-1-ART with various concentrations of DFO for 24 h. To detect mitochondria generated ROS, MitoTracker and MitoSOX were treated. Hoechst were used for nucleus staining. **(B)** Relative fluorescence intensity ratio of MitoSOX/Hoechst of DZ-1-ART ± DFO-treated tumoroids. One-way ANOVA and Tukey’s multiple comparison test were used for statistical analysis (**P < 0.01, ****P < 0. 0001). **(C)** Tumoroids were treated with 10 µM of DZ-1-ART with various concentrations of DFO for 24 h. To detect mitochondria generated apoptosis, TUNEL assay was used. **(D)** Relative fluorescence intensity ratio of TUNEL/Hoechst of DZ-1-ART ± DFO-treated tumoroids. One-way ANOVA and Tukey’s multiple comparison test were used for statistical analysis (*P < 0.05, **P < 0.01, ****P < 0. 0001).

To investigate the role of Fenton reaction–mediated ROS in DZ-1-ART–induced apoptosis, tumoroids were treated with 10 µM DZ-1-ART in the presence of various concentrations of DFO, a Fenton reaction inhibitor. MitoTracker and MitoSOX staining were used to assess mitochondrial ROS. Strong MitoSOX red fluorescence in DZ-1-ART–treated tumoroids confirmed robust mitochondrial ROS generation. In contrast, co-treatment with DFO markedly reduced MitoSOX fluorescence, indicating effective inhibition of DZ-1-ART–induced mitochondrial ROS (Figures 7A,B).

To assess the involvement of mitochondrial ROS in apoptosis, tumoroids were similarly treated with DZ-1-ART ± DFO and analyzed using TUNEL staining. Strong green fluorescence in DZ-1-ART–treated tumoroids confirmed apoptosis induction, whereas DFO co-treatment significantly reduced TUNEL positivity (Figures 7C,D). Collectively, these results demonstrate that DZ-1-ART induces apoptosis in patient-derived tumoroids via Fenton reaction–mediated mitochondrial ROS.

### 3.9 DZ-1-ART induces superoxide mediated ROS and apoptosis in patient-derived tumoroids

**FIGURE 8:**
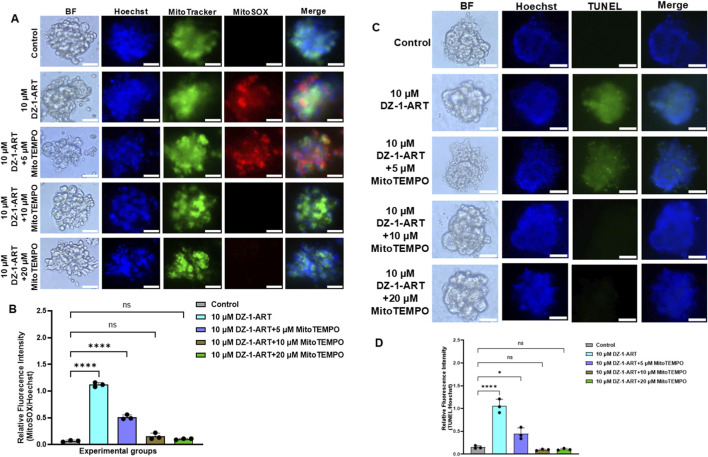
DZ-1-ART induces mitochondria-mediated superoxide ROS generation and apoptosis. **(A)** Tumoroids were treated with 10 µM DZ-1-ART in the presence of varying concentrations of MitoTEMPO for 24 h. Mitochondria-generated ROS were detected using MitoTracker and MitoSOX, and nuclei were stained with Hoechst. **(B)** Relative fluorescence intensity ratio of MitoSOX/Hoechst in DZ-1-ART ± MitoTEMPO-treated tumoroids. Statistical analysis was performed using one-way ANOVA followed by Tukey’s multiple comparison test (****P < 0.0001). **(C)** Tumoroids were treated with 10 µM DZ-1-ART with varying concentrations of MitoTEMPO for 24 h. Mitochondria-mediated apoptosis was assessed by TUNEL assay. **(D)** Relative fluorescence intensity ratio of TUNEL/Hoechst in DZ-1-ART ± MitoTEMPO-treated tumoroids. Statistical analysis was performed using one-way ANOVA followed by Tukey’s multiple comparison test (*P < 0.05, ****P < 0.0001).

To investigate the role of mitochondrial superoxide in DZ-1-ART–induced apoptosis, tumoroids were treated with 10 µM DZ-1-ART in the presence of varying concentrations of MitoTEMPO for 24 h. MitoTracker and MitoSOX staining were used to assess mitochondrial ROS. DZ-1-ART–treated tumoroids exhibited strong MitoSOX red fluorescence, whereas co-treatment with 10 µM or 20 µM MitoTEMPO significantly reduced MitoSOX fluorescence (Figures 8A,B), indicating inhibition of DZ-1-ART–induced mitochondrial superoxide production. Furthermore, TUNEL assays showed markedly reduced green fluorescence in tumoroids co-treated with DZ-1-ART and MitoTEMPO compared to DZ-1-ART alone (Figures 8C,D), confirming that mitochondrial ROS mediates DZ-1-ART–induced apoptosis.

## 4 Discussion

Tumoroids derived from various tumor types have been developed for drug resistance screening and precision therapy (Serra-Camprubí et al., 2023; Paul et al., 2025). Patient-derived tumoroids recapitulate the morphological and cellular characteristics of *in vivo* tumors and closely mimic the tumor microenvironment (TME) (Neal et al., 2018). In contrast, traditional monolayer cell lines are two-dimensional and fail to accurately represent the complexity of actual tumors (Brodeur et al., 2021). In this study, we successfully established colon cancer tumoroids to evaluate the anticancer agent DZ-1-ART.

We have previously demonstrated the tumoricidal efficacy of ART in cancer cells (Hong et al., 2017; Lee et al., 2018; Lee et al., 2019; Lee et al., 2020; Lee et al., 2020; Lee et al., 2025; Kim et al., 2022; Bhowmick and Lee, 2025; Helmueller et al., 2025b; Helmueller et al., 2025a). To preferentially deliver ART to tumor tissue, we synthesized a HMCD DZ-1 dye–conjugated ART derivative, DZ-1-ART. HMCD dyes are known to preferentially target cancer cells due to upregulated OATPs, particularly OATP1B3 and OATP2B1 (Obaidat et al., 2012; Buxhofer-Ausch et al., 2013), as well as elevated HIF-1α expression in tumors (Shi et al., 2014). In earlier work, we showed that DZ-1-ART induces apoptosis via ROS generation in cancer cells (Narayanasamy et al., 2025). In the present study, we determined the IC_50_ concentration of DZ-1-ART to be 10.73 µM (Figure 1A). However, IC50 values obtained from tumoroids may be subject to variability due to the presence of multiple cell types and physical barriers within the 3D structure (Berrouet et al., 2020; Xu et al., 2022). Moreover, a previous study reported that IC50 measurements in patient-derived tumoroids may have limited sensitivity and specificity (Tang et al., 2023). Recently, a Tumoroid-On-a-Plate (ToP) model has been developed to improve drug screening approaches (Seyfoori et al., 2025). We plan to adopt this model in future studies to enhance the accuracy and reliability of our drug response assessments.

Previous studies have demonstrated the involvement of both the extrinsic and intrinsic apoptotic pathways in mediating cytotoxicity in cancer cells (Kim et al., 2009; Winter et al., 2014). We further evaluated the expression of endoplasmic reticulum (ER) oxidative stress–related molecular markers, including HO-1, BiP, and ATF4, in DZ-1-ART–treated tumoroids with or without NAC (Figure 5C). Our results indicate that NAC-mediated attenuation of ER oxidative stress also reduced apoptosis. Consistent with these findings, naphthylchalcones were previously reported to induce apoptosis through concurrent activation of both extrinsic and intrinsic apoptotic pathways in association with ER stress in a leukemic cell line (Winter et al., 2014). Similarly, ER stress may function as a critical link integrating extrinsic and intrinsic apoptotic signaling in DZ-1-ART–treated tumoroids.

We also demonstrate that DZ-1-ART preferentially accumulates in the mitochondria of tumoroids and generates ROS (Figures 3, 5–8). Once inside the cell, DZ-1-ART accumulates in mitochondria due to its positive charge and lipophilic nature. The negative inner mitochondrial membrane potential (∼−180 mV) likely electrostatically attracts and retains this cationic dye. Such mitochondrial accumulation is a common feature of delocalized lipophilic cations, similar to Rhodamine 123 or MitoTracker dyes (Modica-Napolitano and Aprille, 2001).

The mitochondrial localization of DZ-1-ART may induce ROS generation through iron accumulation and Fenton reactions (Thomas et al., 2009). Previous studies have shown that mitochondria-targeted nanoparticles can trigger cell death via Fenton reaction–mediated ROS production in breast cancer cells (Zhong et al., 2022). Because an ester linker is used to conjugate DZ-1 to ART, DZ-1-ART may be cleaved into DZ-1 and ART by mitochondrial esterases. The endoperoxide bridge of cleaved ART can then induce ROS generation, including superoxide and hydroxyl radicals, through iron-mediated Fenton-like reactions. Notably, the mitochondrial Fe^2+^ concentration in tumor cells (20–30 µM) is higher than that in the cytosol (0.five to two µM) (Ni et al., 2020; Tam et al., 2022). The involvement of ART-associated Fenton-like reactions was confirmed using DFO, a strong iron chelator (Figure 7). In this study, we demonstrate that ART effectively generates ROS in the mitochondria (Figures 7, 8). These ROS may oxidize lipids in the mitochondrial membranes through lipid peroxidation, leading to mitochondrial membrane damage, depolarization (reduced Δψm), and dysfunction. Furthermore, the accumulation of mitochondrial superoxide can promote apoptosis (Strathmann et al., 2010; Ma et al., 2011). Using MitoTEMPO, a mitochondria-targeted superoxide dismutase mimetic, we confirmed the role of mitochondrial superoxide radicals (O_2_^−^) in DZ-1-ART–induced apoptosis (Figure 8).

Our studies support the potential of DZ-1-ART as a therapeutic agent for precision oncology. Additional validation using multiple samples would provide greater clarity regarding the selectivity and translational relevance of this study. The limited number of samples represents a major limitation. Therefore, further studies incorporating larger and more diverse sample sets are warranted to confirm the translational relevance of these findings. Another limitation of this study is the absence of *in vivo* validation, which currently restricts direct assessment of translational relevance. However, tumoroid models provide a physiologically relevant intermediate platform that preserves key architectural and molecular features of the native tumor while allowing controlled mechanistic evaluation. Importantly, *in vivo* validation studies are ongoing and will be reported in future work. Future work could involve establishing patient-derived tumoroids to evaluate DZ-1-ART efficacy *ex vivo*. Such personalized assessments may inform the use of DZ-1-ART as a targeted therapy for CRC.

In conclusion, DZ-1-ART efficiently accumulates in patient-derived tumoroids, localizes to mitochondria, disrupts mitochondrial membrane potential, and induces ROS production, ultimately triggering apoptosis. These findings underscore the promise of DZ-1-ART as a precision therapeutic strategy for CRC.

## Data Availability

The original contributions presented in the study are included in the article/supplementary material, further inquiries can be directed to the corresponding author.
